# Enhanced stimulation of anti-breast cancer T cells responses by dendritic cells loaded with poly lactic-co-glycolic acid (PLGA) nanoparticle encapsulated tumor antigens

**DOI:** 10.1186/s13046-016-0444-6

**Published:** 2016-10-26

**Authors:** Soodabeh Iranpour, Vahid Nejati, Nowruz Delirezh, Pouria Biparva, Sadegh Shirian

**Affiliations:** 1 Department of Biology, Faculty of Science, Urmia University, Urmia, Iran; 2 Department of Cellular and Molecular Biotechnology, Institute of Biotechnology, Urmia University, Urmia, Iran; 3 Department of Basic Sciences, Sari Agricultural Sciences and Natural Resources University, Sari, Iran; 4 Department of Pathology, School of Veterinary Medicine, Shahrekord University, Shahrekord, Iran; 5 Shiraz Molecular Pathology Research Center, Dr Daneshbod Pathology Laboratory, Shiraz, Iran

**Keywords:** Dendritic cells, Tumor associated antigen, Nanoparticles, PLGA

## Abstract

**Background:**

Developing safe and effective cancer vaccine formulations is a primary focus in the field of cancer immunotherapy. Dendritic cells (DC) are currently employed as cellular vaccine in clinical trials of tumor immunotherapy. Recognizing the critical role of DCs in initiating anti-tumor immunity has resulted in the development of several strategies that target vaccine antigens to DCs to trigger anti-tumor T cell responses. To increase the efficiency of antigen delivery systems for anti-tumor vaccines, encapsulation of tumor-associated antigens in polymer nanoparticles (NPs) has been established.

**Methods:**

In this study, the effect of tumor lysate antigen obtained from three stage III breast cancer tissues encapsulated within PLGA NPs to enhance the DC maturation was investigated. The T-cell immune response activation was then fallowed up. Fresh breast tumors were initially used to generate tumor lysate antigens containing poly lactic-co-glycolic acid (PLGA) NP. The encapsulation efficiency and release kinetics were profiled. The efficiency of encapsulation was measured using Bradford protein assays measuring the dissolved NPs. The stability of released antigen from NPs was verified using SDS-PAGE. To evaluate the hypothesis that NPs enhances antigen presentation, including soluble tumor lysate, tumor lysate containing NPs and control NPs the efficiency of NP-mediated tumor lysate delivery to DCs was evaluated by assessing CD3+ T-cell stimulation after T cell/and DCs co-culture.

**Results:**

The rate of encapsulation was increased by enhancing the antigen concentration of tumor lysate. However, increasing the antigen concentration diminished the encapsulation efficiency. In addition, higher initial protein contenting NPs led to a greater cumulative release. All three patients released variable amounts of IFN-γ, IL-10, IL-12 and IL-4 in response to re-stimulation. T cells stimulated with lysate-pulsed DCs induced a substantial increase in IFN-γ and IL-12 production. We demonstrated that NPs containing tumor lysate can induce maturation and activation of DCs, as antigen alone does.

**Conclusion:**

PLGA-NPs are attractive vehicles for protein antigen delivery which effectively induce stimulation and maturation of DCs, allowing not only an enhanced antigen processing and immunogenicity or improved antigen stability, but also the targeted delivery and slow release of antigens.

## Background

Breast cancer is the most common malignant tumor and the first leading cause of cancer-related deaths in women worldwide [1]. Although, several methods including surgery, radiotherapy, hormone replacement therapy and chemotherapy have been used to treat breast cancer, an effective treatment for patients with metastatic and invasive breast cancer is yet to be established. Cancer immunotherapy and vaccines are new therapeutic approaches which offer a promising treatment against cancer with minimum adverse effects [2, 3]. Cell-mediated immune mechanisms have been determined in breast cancers [4]. These immune responses are induced by breast tumor cells and thus lead to a systemic anti-tumor immunity and regression of breast cancer [5, 6]. Therefore, immunotherapy may be effective in treating patients with breast cancer. This kind of therapy concentrates on the induction and enhancement of immune responses against tumors. In a limited number of malignancies in which tumor-associated antigens have been determined, antitumor vaccine strategies have proven to be partially effective, predominantly based on the loading of professional antigen-presenting cells (APCs), including DCs [7]. APCs are a group of cells that can process antigens of both endogenous (normal cell proteins, tumor or viral antigens) and exogenous origin (extracellular antigens) [8, 9]. Using DCs which are the most potent APCs with the unique capability to induce primary immune responses against tumor-associated antigens in cancer treatment is a promising approach [10, 11]. In addition, vaccines act through DCs that induce, regulate and maintain T-cell immunity [12]. Ex-vivo loaded DC-based cancer vaccines study has been shown that this strategy is safe, well tolerated and capable of inducing cellular immune responses [13]. DCs and macrophages are highly phagocytic cells, capable of taking up any particles with similar dimensions to the pathogens (up to 10 μm). Therefore, both large “micro-” and small “nano-” particles can efficiently be taken up by both cell types [14].

In clinical medicines, NPs have attracted increasing attention as carriers of therapeutic and/or diagnostic agents [15]. Generally, cellular uptake of NPs is relatively higher than that of microparticles. NPs can entrap a wide range of biologically active compounds varying in their therapeutic indications, such as hormones, antibiotics and anti-cancer drugs [15]. In the recent years, biodegradable polymers, such as Poly (D,L-lactic-co-glycolic acid) (PLGA), have been studied for the fabrication of drug delivery systems and administration of vaccine antigens. PLGA is a FDA approved biodegradable polymer [16] that have been widely used in production of biodegradable surgical sutures and the sustained delivery of drugs into humans [17, 18]. In this study, the effect of breast tumor lysate antigen encapsulated within PLGA NPs to enhance DC maturation and antigen-loaded mature DC stimulated T-cell immune responses against breast cancer have been investigated.

## Methods

### Tumor lysate preparation

Fresh breast tumor was removed from surgical tissues from three breast cancer patients, washed twice in RPMI-1640 (Invitrogen, USA) and dissected into 1–2 mm^3^ pieces under sterile condition. The dissected tissues were incubated overnight at 37 °C in 5 % CO_2_in enzymatic culture medium containing DNase, Hyaloronidase and Collagenease III (1 mg/ml, 0.1 mg/ml and 1 mg/ml, respectively) (Sigma-Aldrich, USA) in order to remove the connective tissue. To remove the undigested connective tissues and cell debris, the resultant cell suspension was centrifuged for 5 min at 200 × *g* and the supernatant was discarded. The cell pallet was washed twice using RPMI 1640 (Sigma-Aldrich, USA) and was resuspended in 1 ml RPMI 1640. Tumor cell lysate was prepared by subjecting the cell suspension to four freeze-thaw cycles (alternating liquid nitrogen and 37 °C water bath treatment) followed by two steps of centrifugation at 300 × *g* for 5 min at 4 °C and then 15,000 rpmfor30 min at 4 °C. The protein concentration of the lysate was measured as described previously [19], the supernatant was then collected and passed through a 0.22 μm filter and stored at −80 °C until use.

### Nanoparticle fabrication

PLGA NPs (Sigma-Aldrich, USA) were fabricated using the solvent evaporation method from a water/oil/water (W2/O/W1) emulsion as described elsewhere [20]. Briefly, PLGA composition (50 % glycolide: 50 % lactide) with inherent viscosity of 0.39 dL/g (Sigma-Aldrich, USA) were dissolved in 2 ml dicholoromethane (DCM) (Sigma-Aldrich, USA). To encapsulate tumor antigen and form a water-in-oil (O/W1) emulsion, three distinct concentrations (15.39, 19.65, 25.86 μg/ml) of the protein solution in PBS (signed as Nanoparticle 1–3) was added to 50 *μl* of organic solution. The emulsion was then sonicated three times for 50s (Soniprep, UK) on ice at a 20 % amplitude. The first emulsion was then made up at three concentrations of 0.5 %, 3 and 5 % by being added drop wise into a 20 ml solution of poly vinyl alcohol (PVA) (Sigma-Aldrich, USA) in a glass test tube and sonicated simultaneously. After sonication, thesecond emulsion, W2/O/W1 emulsion, was poured into a beaker containing 50 ml of 0.25 % PVA followed by sonication for 10 s. To eliminate organic solvent, the second emulsion was then stirred at 500 rpm and kept under laminar air flow hood overnight. The NP slurry was then centrifuged at 16,000 rpm for 40 min to be sedimented. The NPs were then washed three successive times with 10 ml of distilled water to remove unentrapped peptides, residual PVA surfactant and large particles. Finally, resultant NPs were resuspended in 5 ml of water and frozen at −20 °C before being lyophilized.

### Nanoparticle characterization

Scanning electron microscopy (SEM) was employed to characterize NPs in terms of size and morphology. A thin film of test samples was deposited onto a metal stub with double-sided adhesive carbon tape (Nisshin EM. Co. Ltd., Tokyo, Japan) and coated with a thin layer of gold for visualization by SEM. Images were collected at three magnifications (20,000, 10,000 and 4000) and analyzed with the DigXY program; a representative sampling of NP diameters was recorded and analyzed for each treatment.

### Encapsulation efficiency measurement

To determine the encapsulation efficiency, 5 mg of lyophilized NP was dissolved in 500 μl of DCM (Sigma-Aldrich, USA) to degrade the NPs. After degradation, 100 μl PBS was gently added to the solution and vortexed three times, each time for 10 s, to increase the contact surface between hydrophilic materials including PBS and peptides. Supernatant of the samples were collected and analyzed for total protein concentration using Bradford assay (Biometer, Germany). The bovine serum albumin (BSA) concentrations used as the standard ranged between 0.5 and 250 μg/ml. Finally, the encapsulation efficiency was calculated using the following formula as described by Prasad et al. [21]:

$$ \mathrm{Protein}\ \mathrm{released}/\mathrm{total}\ \mathrm{protein}\ \mathrm{content}\ \mathrm{of}\ \mathrm{N}\mathrm{P}\mathrm{s} \times 100 $$

### Release rate measurement

The rate of protein release from NPs was measured under controlled condition. Five milligrams of NPs were dispersed in 500 μl PBS, pH 7.4, and the suspension was incubated at 37 °C with continuous agitation in an orbital shaker (200 rpm/min). Periodically, the suspension was centrifuged at 15,000 × *g* for 5 min to pellet the spheres. Supernatants were then collected and stored at −20 °C for later analysis. The spheres were serially resuspended in 500 μl of fresh PBS in the original tube for further incubation. The concentration of the released protein was measured using Bradford assay, applying BSA as the standard. The cumulative release was studied for 7 days and the release efficiency was calculated as follow:

$$ \mathrm{Released}\ \mathrm{protein}/\ \mathrm{total}\ \mathrm{protein}\ \mathrm{content}\ \mathrm{of}\ \mathrm{N}\mathrm{P}\mathrm{s} \times 100 $$

### Estimating protein composition and integrity

To determine whether a representative selection of tumor-associated proteins have been encapsulated and released, protein composition before and after encapsulation were compared on the 10 % gradient SDS-PAGE by Silver Xpress staining kit (Invitrogen, USA).

### Dendritic cell generation

Dendritic cells were generated from human peripheral blood mononuclear cells (PBMCs). Whole blood of each patient with breast cancer was collected in separate heparin-containing 50 ml conical tubes and diluted 1:1 in sterile PBS, layered on ficoll/Hypaque (lymphodex, Germany) solution, and centrifuged at 1000 *g* for 30 min at 25 °C. In order to deplete the platelets, the cells were collected and washed with RPMI 1640 (Sigma-Aldrich, USA) twice and PBMCs were cultured in RPMI-1640 supplemented with heat-inactivated filter-sterilized 10 % (v/v) human AB^+^ serum, 100U/ml penicillin, and 100 μg/ml streptomycin (Sigma-Aldrich, USA) with seeding density of 2 × 10^6^cell/ml. The cultured cells were then incubated for 2 h at 37 °C and 5 % CO_2_ to provide the condition for adhering the monocytes. Non-adherent cells were then removed, adherent cells were kept in RPMI-1640 (Sigma-Aldrich, USA) containing 1000U/ml granulocyte-macrophage colony-stimulating factor (GM-CSF) (Sandoz Basel, Switzerland) and 800U/ml IL-4 (Peprotech, Germany). The culture medium was changed on days 3, 5 and 6. On day 3, the same amount of cytokines was added to the culture medium and immature DCs were then pulsed with NPs, tumor lysate and tumor lysate loaded NPs (NP-tumor lysate) 0.2 mg per 1 × 10^6^ cells, and were incubated for further 24 h. On day 5, the same volume of culture media was substituted by monocyte conditioned medium (MCM) (25%V/V) and 20 ng/ml TNF-α (eBioscience, USA) was added as maturation factors; the DCs were harvested after 48 h and subjected to immunophenotyping. The supernatant was collected and stored at −20 ˚C for IL-10 and IL-12 cytokines analysis.

### Immunophenotyping of monocyte-derived DCs

Immunophenotyping of monocyte-derived DCs was performed by direct immunofluorescence staining of cell surface markers using FITC-labeled anti-CD14, CD83, HLA-DR, CD86, CD80 and the appropriate isotype controls (Serotech, UK). Samples were analyzed on FACScanCalibur (Becton Dickinson, USA) using Cell Quest software.

### T cell proliferation assay

The efficiency of NP-mediated tumor lysate delivery into DCs was established by assessing CD3+ T-cell stimulation after co-culturing autologous T cells and DCs. CD3^+^ T cells were purified positively from PBMCs by anti CD3 microbead according to manufacturer’s protocol (Miltenyi, Germany). The purity of T cells was more than 90 %, which was determined by monoclonal anti-CD3 antibody staining. Isolated T cells were co-cultured with antigen loaded DCs, in 200 μl complete medium (RPMI 1640 supplemented with 10 % AB serum, 1 % HEPES, 100 U/ml penicillin and 100 μg/ml streptomycin) at a seeding density of 4 × 10^5^ cells per well in 96-well flat bottom microtiter plate. The DC:T ratio was1:5, 1:10, 1:20. The cultured cells were incubated for 5 days at 37 °C in 5 % CO_2_. Phytohemagglutinin (PHA) stimulated T cells (2.5 %) (Sigma-Aldrich, Germany) and DC or T cells alone were used as positive and negative controls, respectively. T cell proliferation rate was measured using the 3-(4, 5-dimethylthiazoyl-2-yl)-2,5-diphenyltetrazolium bromide (MTT) assay. The assay reflected the activity of mitochondrial dehydrogenase that transforms light yellow MTT into dark blue formazan. 20 μl of 5 mg/ml MTT solution was added into each well for 5 day coculture of T: DC and the plates were then incubated at 37 °C for 4 h in a 5 % CO_2_supplied incubator. The culture medium was removed and the formazan crystals were dissolved in Dimethyl sulfoxide (DMSO). The absorbance of formazan solution was measured at 570 nm using an ELISA reader (Awerness, USA), and proliferation index was measured as follow:

$$ \mathrm{Proliferation}\ \mathrm{Index}=\frac{\mathrm{Sample}\ \left(\mathrm{O}\mathrm{D}\right)\ \hbox{--}\ \mathrm{Blank}\ \left(\mathrm{O}\mathrm{D}\right)}{\mathrm{Control}\ \left(\mathrm{O}\mathrm{D}\right)\ \hbox{--}\ \mathrm{Blank}\ \left(\mathrm{O}\mathrm{D}\right)} $$

### Cytokine release assay

Finally, IL-10 and IL-12 cytokine released into supernatant of mature DCs and IFN-ϒ and IL-4 content of the stimulated T cell supernatants were analyzed using commercially available ELISA kits (Peprotech, USA).

### Statistical analysis

Statistical analysis was done using SPSS version 17.0 (SPSS, Surrey UK). Mann–Whitney U and Kruskal-Wallis tests were used to compare cytokine production by DC and T cells under different conditions. The data depicted in each figure corresponds to one representative experiment of at least three independently performed experiments. A level of ≤ 0.05 was deemed to be statistically significant.

## Results

### Encapsulation efficacy and release measurement

**Fig. 1:**
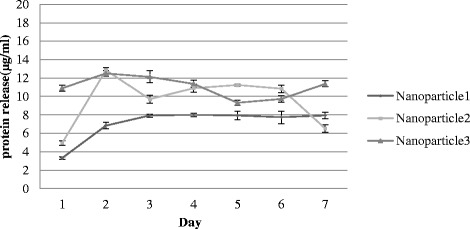
Comparison of cumulative protein release from nanoparticles fabricated from freshly isolated human breast cancer tumor lysates. The amount of protein released from nanoparticle 1–3 was 9.93 μg/mg, 13.40 μg/mg and 15.66 μg/mg, respectively

Encapsulation efficiency (EE) of the tumor lysate into NPs was measured by calculating the ratio of the released proteins to the initial amount of tumor extract, as explained in method section. The rate of encapsulation was increased with enhancing the protein concentration of tumor lysate which resulted in a decrease in the encapsulation efficacy (Table 1). Over a 7-day period, approximate functional life span of an Ag-loaded DC, cumulative release of NP was measured during incubation of NP phosphate buffered saline to estimate the total amount of Ag delivered from NPs. Biphasic protein release, “burst of protein release”, was observed over the first 24–48 h and a further sustained release was not observed over the period of the subsequent days (Fig. 1). In addition, higher initial protein content in the NPs led to a greater cumulative release (Table 2).

**Table 1:** Effect of 3 different concentration of tumor- derived lysate on encapsulation rate and encapsulation efficiency

Samples	Tumor lysate protein concentration (mg)	Volume of tumor lysate (μl)	Tumor lysate encapsulation rate (μg/mg)	PLGA amount for encapsulation (mg)	Encapsulation efficiency (EE%)
Nanoparticle1	15.39	50	12.31	50	79.9
Nanoparticle2	19.65	50	16.4	50	83.46
Nanoparticle3	25.86	50	18.8	50	72.69

**Table 2:** Effect of poly lactic-co-glycolic acid (PLGA) protein content on release rate

Samples	Protein content encapsulated in PLGA (μg/mg)	Release content (μg/mg)
Nanoparticle1	12.31	9.93
Nanoparticle2	16.4	13.40
Nanoparticle3	18.8	15.66

### Conservation of the entrapped antigen integrity

**Fig. 2:**
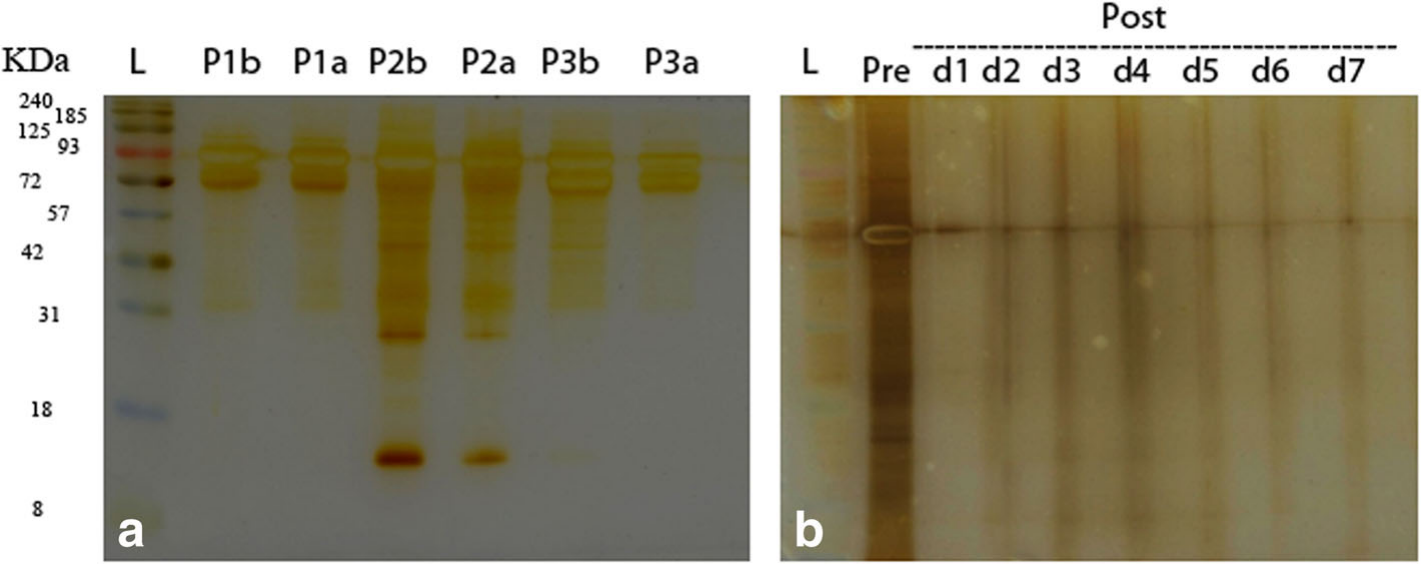
Silver-stained SDS–PAGE analysis of tumor lysates in PLGA nanoparticle supernatant before and after encapsulation. **a** Proteins contents of tumor lysates before and after encapsulation from three patients (*n* = 3), **b** Protein contents of tumor lysate before encapsulation (pre) and seven successive days after encapsulation (post 1–7), L: protein ladder

To verify the possible changes in tumor-associated protein contents during encapsulation process pre- and post-encapsulation samples were subjected to electrophoretic analysis on SDS-PAGE gels followed by silver-staining. A relative paucity of bands between 10 and 70 kDa was noted in NP released samples with silver-stained SDS-PAGE gel (Fig. 2a). Pre and post encapsulation protein release indicated and uniform molecular weight and bands during over 7 days or an approximate functional life span of an Ag-loaded DC (Fig. 2b).

### Morphology and size

**Fig. 3:**
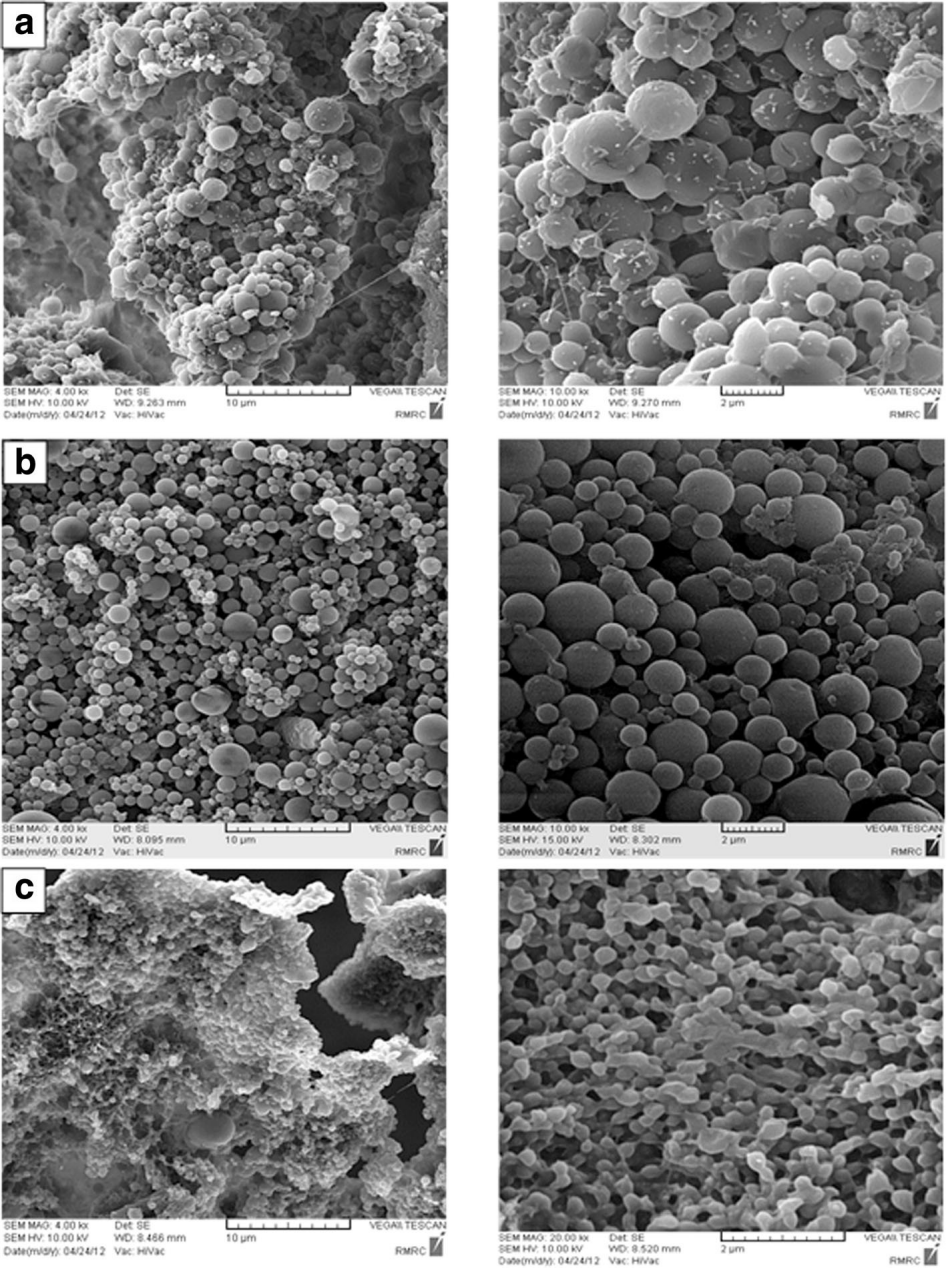
Scanning electron micrograph of PLGA 50:50, 75KDa manufactured with various concentrations of PVA; **a** 0.5 % PVA, **b** 3 % PVA and **c** 5 % PVA. NPs formed with higher concentration of PVA were smaller than those made using lower concentration of PVA (Mean ± SD: 232 ± 0.62 nm vs. 1062 ± 4.65 nm)

To measure the morphology and the size of the antigen loaded NPs, three different concentrations of PVA were used as a stabilizer (0.5, 3 and 5 %). Scanning electron microscopy was used to compare morphological appearances of the NPs derived from soluble lysates. All NP preparations were spherical with a wide size distribution. The smallest range of NPs was detected in the highest concentration of PVA (Fig. 3).

### Biological activity of encapsulated antigen

**Fig. 4:**
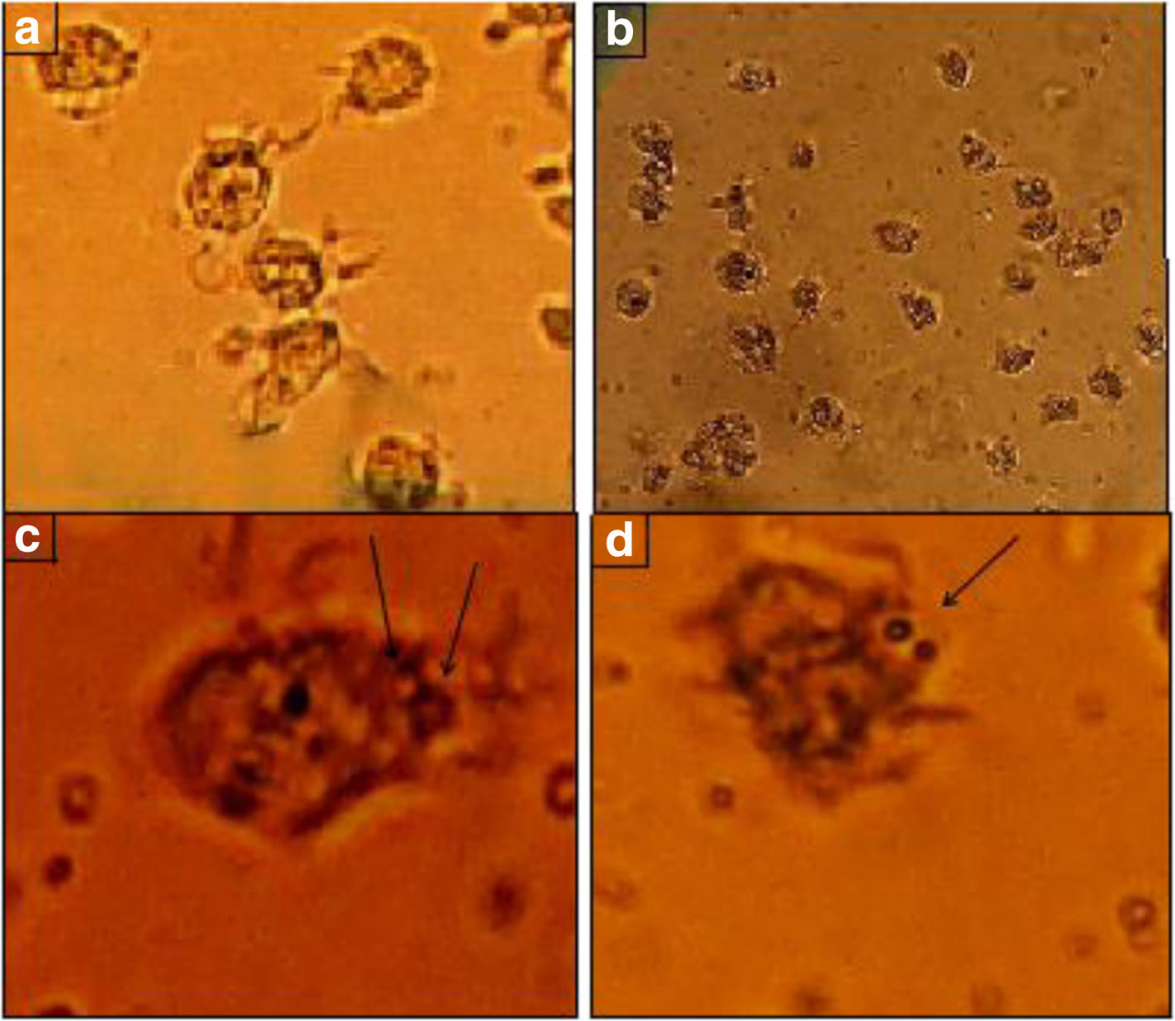
Phagocytosis of tumor antigen pulsed nanoparticles by immature dendritic cells; **a** immature dendritic cells on day 4, **b** immature dendritic cells treated with nanoparticles, **c** and **d** nanoparticles associated with the cells and internalized (*black arrows*)

To determine the effectiveness of NP mediated antigen delivery, a series of experiments were performed using monocyte-derived DCs and peripheral blood CD3+ T cells. The efficiency and rate of encapsulation were evaluated in three concentrations, including 15, 20, and 25 μg/ml of tumor lysate. The data showed that the efficiency and rate of encapsulation at intermediate concentration (20 μg/ml) of NPs were at their highest. Tumor lysate with concentration of 120μg/ml without any PLGA and NPs alone were used as control (Fig. 4).

### Immunophenotyping of DCs

**Fig. 5:**
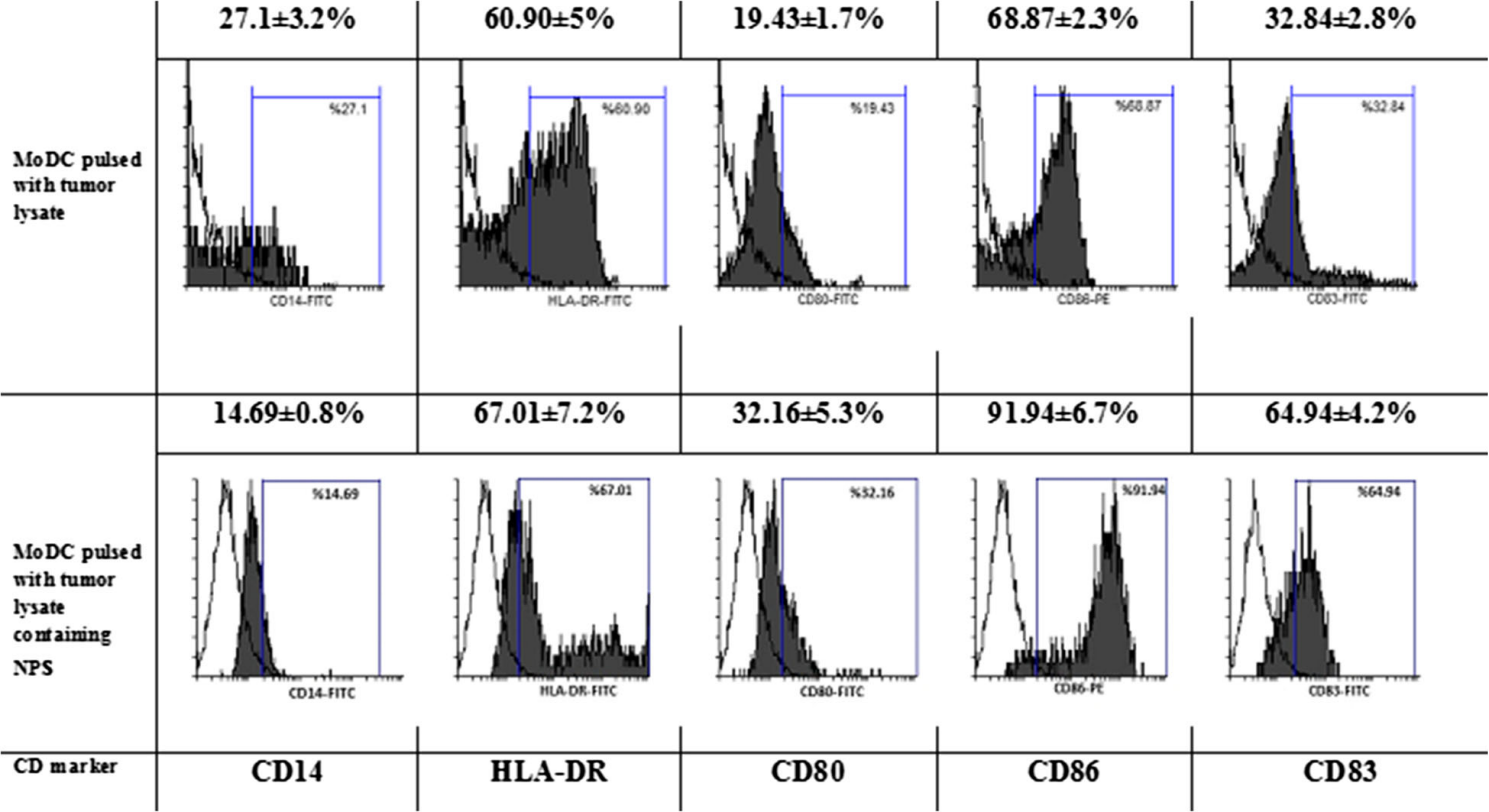
Representative chart of flow cytometric analysis of monocyte-derived DCs. DCs were harvested on day 7 and analyzed by flow cytometry using monoclonal anti-CD14, HLA-DR, CD80, CD86 and CD83 antibodies and respective isotype controls. Flow cytometric analysis of DCs treated with NP-containing antigen showed that expression of CD80, CD86, HLADR, CD83 was up-regulated, while expression of CD14 surface marker was down-regulated in comparison to DCs pulsed with tumor lysate alone (*P* ≤ 0.05) Depicted data are averaged from three independent samples obtained from breast cancer patient (*n* = 3) (*: indicates the statistical significance)

**Fig. 6:**
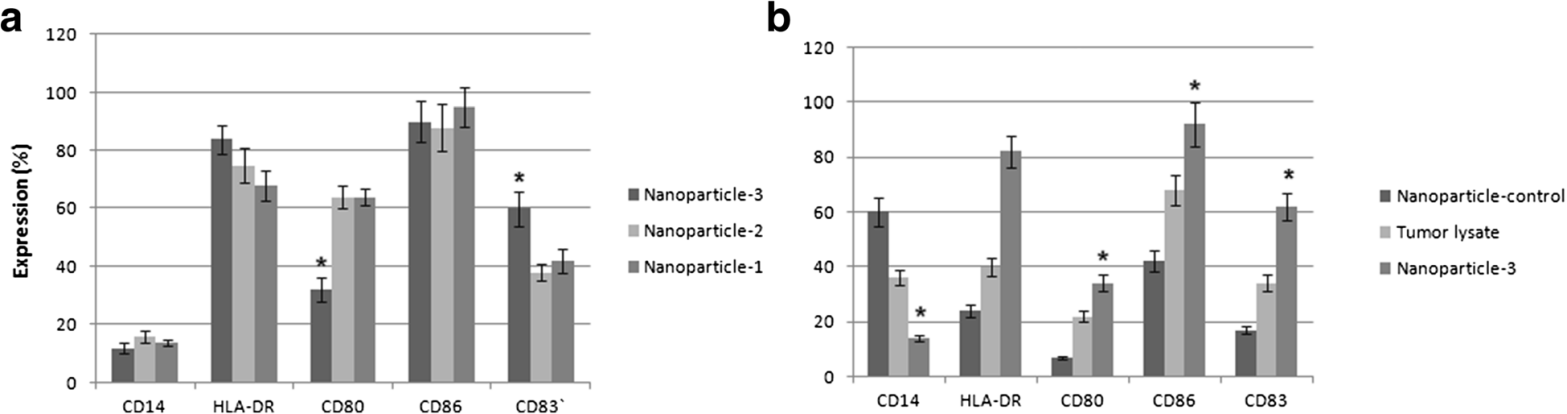
Immunophenotyping of monocyte derived dendritic cells pulsed with either tumor lysate or tumor lysate containing nanoparticles, **a** DCs treated with tumor antigen pulsed nanoparticles fabricated using three different amounts (concentrations) of PVA, Our results showed that NP3 pulsed DCs expressed upper levels of, HLA-DR and lower levels of CD14 and CD86 markers significantly (*P* ≤ 0.05), while expression of CD83 and CD80 markers were significantly up and down-regulated respectively (*P* ≤ 0.05). **b** Comparison of DCs pulsed with nanoparticle containing tumor antigen with those pulsed with either tumor lysate or NPs alone, the data of this study showed that the tumor lysate loaded NPs triggers a more efficient maturation of monocyte-derived DCs compared to either tumor lysate or NPs alone (*P* ≤ 0.05) Depicted data are averaged from three independent samples obtained from breast cancer patients (*n* = 3) (*: indicates the statistical significance)

Flow cytometric analysis of DCs treated with three different manufactured antigen loaded NPs compared to either tumor lysate or NPs alone showed thatNP3 pulsed DCs expressed upper levels of, HLA-DR and lower levels of CD14 and CD86markers significantly (*P* ≤ 0.05), while expression of CD83 and CD80 markers were significantly up and down-regulated respectively (*P* ≤ 0.05). Furthermore, the data of this study showed that the tumor lysate loaded NPs triggers a more efficient maturation of monocyte-derived DCs compared to either tumor lysate or NPs alone (*P* ≤ 0.05) (Figs. 5 and 6).

### T cell proliferation

**Fig. 7:**
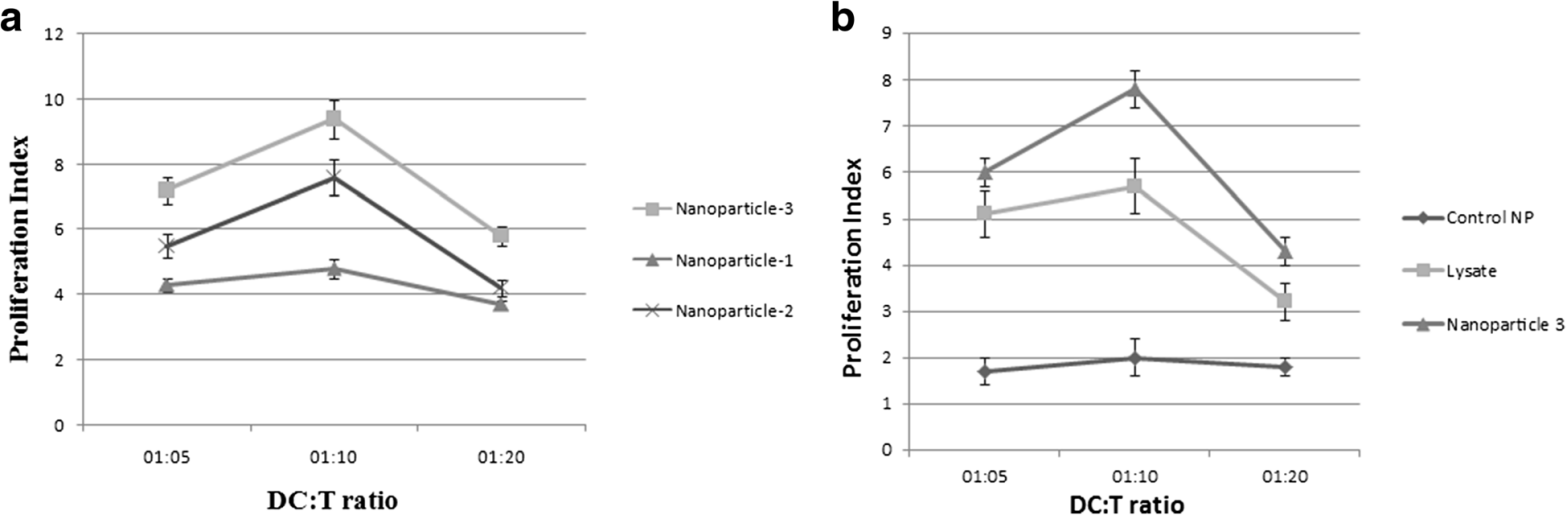
T cells proliferation responses induced by dendritic cells. Magnetic cell sorting (MACS)-enriched CD3+ T cells were stimulated with autologous DCs pulsed with **a** DCs treated with tumor antigen pulsed nanoparticles fabricated using three different amounts (concentrations) of PVA, **b** Comparison of DCs pulsed with nanoparticles containing tumor antigen with those pulsed with either tumor lysate or NPs alone, at a ratios of 5:1, 10:1 and 20:1 for five days and proliferation rates were meassured by MTT assay. The triplicate proliferation indices from three patients were averraged and expressed as mean ±S.D (*n* = 3) (*P* 0.05)

CD3+ T-cell populations were isolated by magnetic cell sorting (MACS); DCs were generated from peripheral blood using granulocyte-macrophage colony-stimulating factor (GM-CSF) and IL-4. Loaded DCs were used to stimulate autologous CD3+ T cells in the standard co-culture experiments. After 5 days of interaction, supernatants of co-incubated DCs and CD3+ T cells were collected and analyzed for cytokine production and MTT assay was used to measure stimulation index and NP-mediated antigen delivery induced T cell proliferation, in three kinds of fabricated NPs and respective ratios for all three patients. Although, proliferation rate and stimulation index varied from patient to patient, however NP3 showed highest proliferation indices in all three patients. The highest proliferation rate was detected in 1:10 ratio of DCs and T cells (Fig. 7a). Furthermore, in comparison to the control groups (tumor lysate and NP alone) higher T cell stimulation was observed in the tumor antigen loaded NP3 nonsignificantly (Fig. 7b).

### Cytokine release

**Fig. 8:**
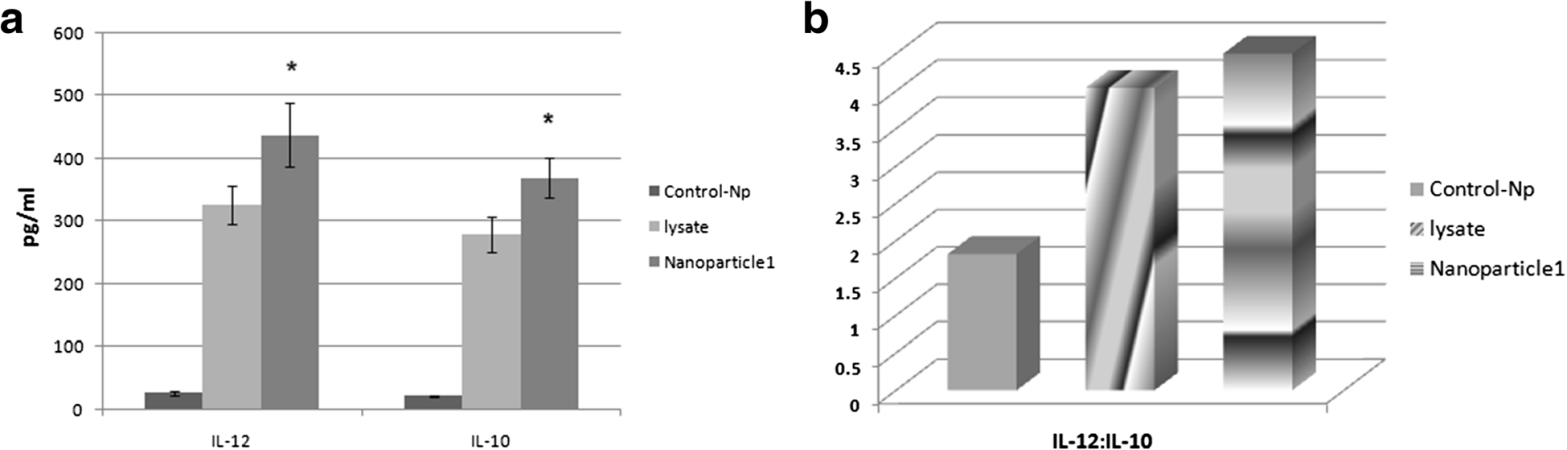
Cytokines released by mature DCs in the supernatant of seventh day of DC culture. IL-12 and IL-10 production were measured using commercially available ELISA kit. Triplicate data obtained from three patients were averaged and expressed as mean ± S.D (*n* = 3). **a** cytokines produced by mature DCs, **b** IL-12:IL-10 ratio, (*P* ≤ 0.05) (*: indicates the statistical significance)

IL-12 and IL-10 cytokine profile of mature DCs was analyzed in the supernatant of seventh day DC culture using a commercially available sandwich ELISA. In comparison to tumor lysate alone, tumor antigen loaded NP pulsed DCs substantially released more IL-12 and IL-10 cytokines, moreover, our findings also revealed that the level of secreted IL-12 was higher than of IL-10 significantly (*P* ≤ 0.05) (Fig. 8a, b).

**Fig. 9:**
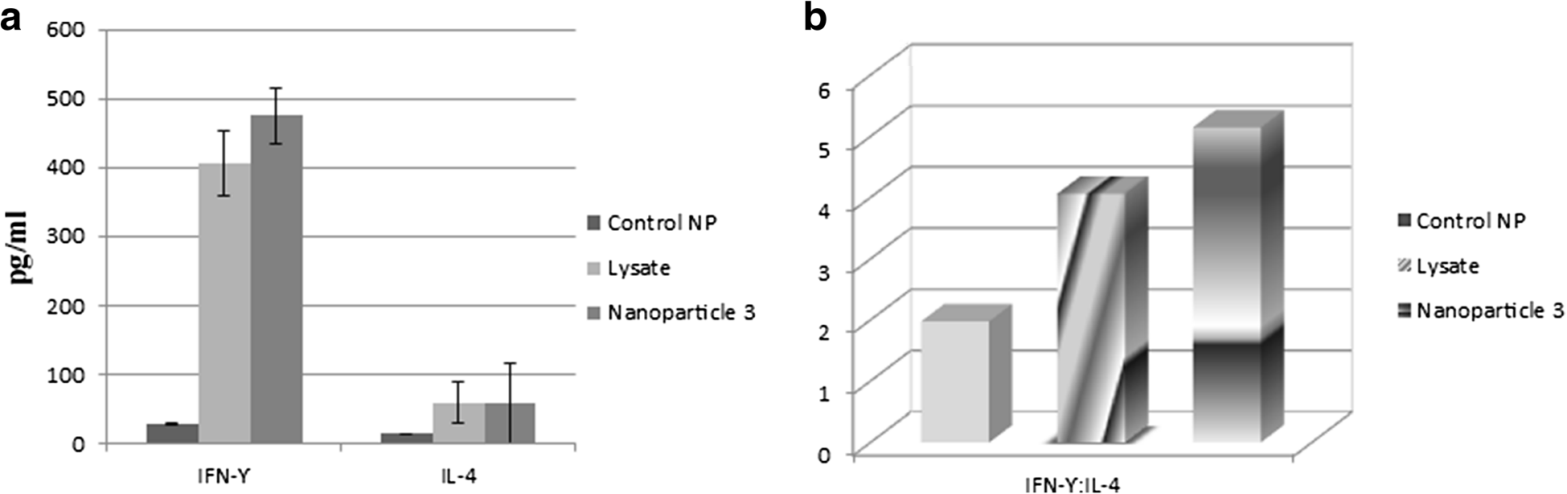
Cytokines released by autologous CD3 + T-cells stimulated with tumor antigen pulsed dendritic cell. Magnetic assay cell sorting (MACS)-enriched CD3^+^T cells were stimulated with autologous DCs pulsed with NP-containing tumor lysate as well as tumor lysate alone and untreated control-NPs for 5 days and IFN-γ and IL-4 production were measured using commercially available ELISA kit. Triplicate data obtained from three patients were averaged and expressed as mean ± S.D (*n* = 3). **a** cytokines produced by T cells, **b** IFN-γ:IL-4 ratio

Encapsulated lysate pulsed DCs stimulated T cells of all three patients released variable amounts of IFN-γ and IL-4, compared to tumor lysate alone, stimulation of T cells with NP-lysate-pulsed DCs induced a nonsignificant increase in IFN-γ and IL-4secretions (Fig. 9a, b).

## Discussion

In the present study, the effect of PLGA NPs in delivery of tumor antigen to DCs has been evaluated. A wide variety of NPs have been developed and employed as delivery vehicles in the form of micro or nanoparticles. PLGA as a biodegradable particle have been successfully used as vehicles for antigen delivery [20]. NPs enhance not only the immunogenicity and stability, but also the targeted delivery and slow release of antigens. DCs as specialized APCs are currently of particular interest in cancer immunotherapy. However, there are a few studies have been carried out to evaluate encapsulation of the tumor lysate derived from solid-organ malignancies within PLGA spheres in humans [21]. Encapsulation and delivery of an antigen mixture derived from breast cancer in human has not yet been reported. The aims of investigating the encapsulation techniques for tumor lysates in murine and human models are to enhance the delivery of tumor associated proteins into the microenvironment of DC maturation and graduate release of antigens within the approximate period of DC functional maturation or life span [20, 21]. The findings of the present study showed that increase in concentration of tumor lysate led to an increment in the rate of encapsulation or protein entrapment. In contrast, the encapsulation efficiency was decreased at higher concentrations of tumor lysate. This reduction in encapsulation efficiency is likely due to the higher protein concentration gradient from the inner to the outer aqueous phase [22]. This pattern of encapsulation has been also reported by some other studies [21, 23]. The data in the present study illustrated that the optimal concentration of NPs to achieve the best efficiency and rate of encapsulation was the intermediate concentration; (20 μg/ml). This concentration of polymer might enhance the protein encapsulation as the result of faster solidification of the particles. Antigen cross-presentation to CD8^+^Tcells was found to be low, when delivered in soluble form. However, encapsulation within PLGA NPs increased cellular uptake of soluble antigen and led to a 1000-fold increase in T-cell mediated immunostimulatory cytokine secretion compared to the antigen-free loafing. It has been also demonstrated in this study that the higher concentration of initial protein loaded in the NPs produced a greater burst release. Furthermore, it has been shown that particle porosity of PLGA is increased by higher concentration of initial loaded tumor lysate, which facilitates diffusion of proteins and leads to a faster release of proteins [22]. It has been reported that the rate of protein release increases with an increment in protein or antigen content of the NPs at a constant polymer molecular weight. The fast release rate is attributed to the higher concentration gradient between the antigen-rich NPs and the outer water phase [24]. Based on the results of this study, burst of protein release was observed over the first 24–48 h and a further sustained release was not observed over the period of the subsequent days. This could be due to the increased encapsulation and release of higher molecular weight proteins. The biphasic release pattern, in which a short burst is followed by a period of linear release, may be particularly useful in antigen delivery to DCs [20], since it provides a continuous supply of antigen complexes on the DC surface for cytotoxic T lymphocytes stimulation [25]. It has been mentioned that low-burst release of encapsulated antigen is crucial for an efficient MHC class I antigen presentation and CD8+ T cell activation [26].

Decreased immunoinhibitory cytokines IL-10 and IL-4 or increased immunostimulatory cytokines IL-12 and IFN-γ cytokines was detected in cells obtained from all of three patients after PLGA-mediated antigen delivery. The secretion level of IFN- γ was higher than that of IL-4 or IL-12 than IL-10 in all three patients. Regarding literature review and our results as well, it is clear that in comparison to tumor lysate alone, PLGA-NP mediated breast tumor antigen delivery to DCs did not result in significant differences in either IFN-γ or IL-4 production as well as immune response polarization, but in the case of PLGA-NP mediated antigen delivery when the tumor lysate is delivered via PLGA-NP lower amounts of antigen is significantly required to elicit same magnetitude of immune responses. This is very important in the cases where the tumor antigen mass is limited when the antigen scours is fine needle biopsies or metastatic sites of tumor.

Therapeutic vaccination is given following the onset of disease and aims to activate and maturate the DCs and macrophages, and in turn, expansion of cell mediated cytotoxicity which eventually leads to death of tumor cells. Matured DCs secrete the T-cell differentiation factor, IL-12, which present antigens more effectively and up-regulate co-stimulatory molecules as a result of increased phenotypic stability and extended half-life of MHC class I- and II molecules [27]. Once a cell-mediated immune response is propagated, cytokines (IFN- γ and TNF-α) and chemokines along with contact-mediated cytotoxicity result in death of the tumor cells [28].

Although, in the most of the previous studies tumor cell lines expressing the immunodominant peptides have been used for encapsulation along with tailored T-cell lines for certain specific epitopes, there is one study in which the whole-tumor material from freshly excised surgical specimens and freshly isolated T cells had been used as it has been done in the present study [21].

Unlike previous studies, no variations in cytokine profiles among samples from three different patients have been observed in the present study. NPs delivery resulted in a greater immuno-stimulatory secretion of IFN-γ and adversely lower immunoinhibitory secretion of IL-10 in all three patients. NPs encapsulated with tumor lysate were able to stimulate specific T cells to produce larger quantities of Th1 and Th2-based cytokines including IFN- γ and IL-4, respectively. The higher concentrations of IFN- γ and IL-12 were detected in NPs encapsulated tumor lysate compared to tumor lysate and NPs alone. The results of this study provide evidence of principle that the whole tumor lysates can stimulate T cell-released cytokines, when delivered to DC in a particulate form. Up-regulationin expression of CD80, CD86, HLADR and CD83 markers and down-regulation in expression of CD14surface marker showed that the incubation of immature DCs with both NP containing antigen and antigen alone would lead to the phenotypic maturation and activation of DCs. Antigens or pathogens and pathogen-associated molecules can naturally induce DC maturation [29]. It has also been reported that PLGA-MS-treated and, subsequently, matured DCs display the same strong enhancement in their capacity to stimulate naive autologousT helper cells and secret the same amount of the cytokines, such as IL-12, IL-10 and TNF.

## Conclusions

PLGA-NPs are attractive vehicles for protein antigen delivery for an effective stimulation and maturation of DCs, allowing not only an enhancement in antigen processing and immunogenicity or improvement of antigen stability, but also they improve the targeted delivery and slow release of antigens. Matured and stimulated DCs display the strong enhancement in their capacity to stimulate naive autologous T helper cells and secret the amount of the cytokines.

## Abbreviations

Ag: Antigen
APCs: Antigen-presenting cells
BSA: Bovine serum albumin
DC: Dentritic cell
DCM: Dicholoromethane
DMSO: Dimethyl sulfoxide
GM-CSF: Granulocyte-macrophage colony-stimulating factor
IFN-γ: Gamma interferon
IL-10: Interleukin-10
IL-12: Interleukin-12
IL-4: Interleukin-4
MCM: Monocyte conditioned medium
MTT: 3-(4, 5-dimethylthiazoyl-2-yl)-2,5-diphenyltetrazolium bromide
NP: Nanoparticle
PBMCs: Peripheral blood mononuclear cells
PBS: Phosphate buffered saline
PHA: Phytohemagglutinin
PLGA: Poly lactic-co-glycolic acid antigen
PVA: Poly vinyl alcohol
SDS-PAGE: SDS-polyacrylamide gel electrophoresis
SEM: Scanning electron microscopy
TNF-α: Tumor necrosis factor-alpha
W2/O/W1: Water/oil/water
